# RETRACTED: Pérez-Albacete Martínez et al. Evaluation of a New Dental Implant Cervical Design in Comparison with a Conventional Design in an Experimental American Foxhound Model. *Materials* 2018, *11*, 462

**DOI:** 10.3390/ma19081559

**Published:** 2026-04-14

**Authors:** Maria Ángeles Pérez-Albacete Martínez, Carlos Pérez-Albacete Martínez, José Eduardo Maté Sánchez De Val, María Luisa Ramos Oltra, Manuel Fernández Domínguez, Jose Luis Calvo Guirado

**Affiliations:** 1 International Dentistry Research Cathedra, Universidad Católica San Antonio de Murcia (UCAM), Av. Jerónimos, 135, 30107 Guadalupe, Murcia, Spain; maperez6@alu.ucam.edu; 2 Oral and Implants Dentistry Department, International Dentistry Research Cathedra, Faculty of Health Sciences, Universidad Católica San Antonio de Murcia (UCAM), Av. Jerónimos, 135, 30107 Guadalupe, Murcia, Spain; mlramos@ucam.edu (M.L.R.O.); jlcalvo@ucam.edu (J.L.C.G.); 3 Oral and Implants Dentistry Department, Biomaterials Research Cathedra, Faculty of Health Sciences, Universidad Católica San Antonio de Murcia (UCAM), Av. Jerónimos, 135, 30107 Guadalupe, Murcia, Spain; jemate@ucam.edu; 4 Department of Oral and Implant Dentistry, Faculty of Dentistry, Universidad San Pablo CEU, Grupo HM (Hospital Madrid), 11600 Madrid, Spain; clinferfun@yahoo.es

The journal retracts the article “Evaluation of a New Dental Implant Cervical Design in Comparison with a Conventional Design in an Experimental American Foxhound Model” [1], cited above.

Following publication, concerns were brought to the attention of the Editorial Office regarding the integrity of an image presented in this publication [1].

In accordance with the journal standard procedures, an investigation was conducted by the Editorial Office and Editorial Board which identified an overlap between Figure 3 in [1] and Figure 2 [2], from a previously published (subsequently retracted) article, produced by a similar authorship group presenting different experimental conditions. In addition, unusual similarities between the results presented in Table 2 in [1] and those reported in Table 1 in [2] were also identified. While the authors collaborated within this process, raw material meeting the journal’s requirements for original images could not be provided for Editorial Board evaluation. Consequently, the Editorial Board has lost confidence in the reliability of the findings and has decided to retract this publication, as per MDPI’s retraction policy.

This retraction was approved by the Editor-in-Chief of the journal *Materials*.

The authors have been informed of this retraction. Carlos Pérez-Albacete Martínez has indicated his agreement with this decision. The remaining authors did not provide a comment on this decision.
